# Electrocatalyst Microenvironment Engineering for Enhanced Product Selectivity in Carbon Dioxide and Nitrogen Reduction Reactions

**DOI:** 10.1021/acscatal.3c00201

**Published:** 2023-04-06

**Authors:** Huali Wu, Amrita Singh-Morgan, Kun Qi, Zhiyuan Zeng, Victor Mougel, Damien Voiry

**Affiliations:** † Institut Européen des Membranes, IEM, UMR 5635, Université Montpellier, ENSCM, CNRS, Montpellier 34000, France; ‡ Department of Chemistry and Applied Biosciences, ETH Zürich, Zürich 8093, Switzerland; § Department of Materials Science and Engineering, City University of Hong Kong, Kowloon, Hong Kong, P. R. China

**Keywords:** carbon dioxide, nitrogen, electrochemical reduction, microenvironment, selectivity, electrocatalyst, electrolyte, three-phase interface

## Abstract

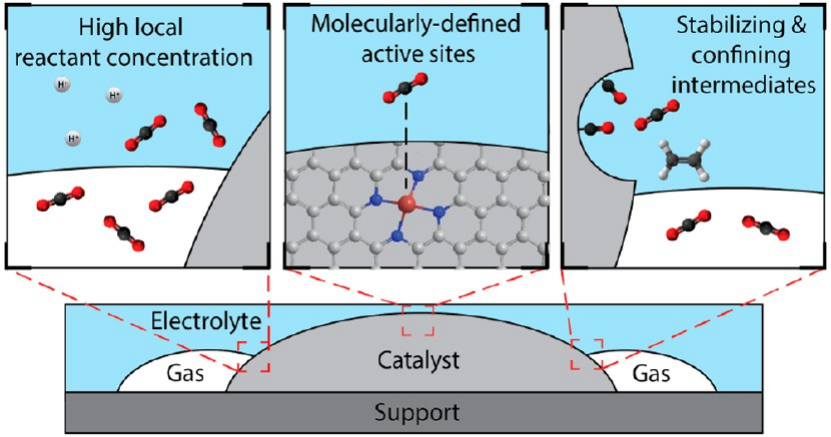

Carbon and nitrogen fixation strategies are regarded as alternative routes to produce valuable chemicals used as energy carriers and fertilizers that are traditionally obtained from unsustainable and energy-intensive coal gasification (CO and CH_4_), Fischer–Tropsch (C_2_H_4_), and Haber–Bosch (NH_3_) processes. Recently, the electrocatalytic CO_2_ reduction reaction (CO_2_RR) and N_2_ reduction reaction (NRR) have received tremendous attention, with the merits of being both efficient strategies to store renewable electricity while providing alternative preparation routes to fossil-fuel-driven reactions. To date, the development of the CO_2_RR and NRR processes is primarily hindered by the competitive hydrogen evolution reaction (HER); however, the corresponding strategies for inhibiting this undesired side reaction are still quite limited. Considering such complex reactions involve three gas–liquid–solid phases and successive proton-coupled electron transfers, it appears meaningful to review the current strategies for improving product selectivity in light of their respective reaction mechanisms, kinetics, and thermodynamics. By examining the developments and understanding in catalyst design, electrolyte engineering, and three-phase interface modulation, we discuss three key strategies for improving product selectivity for the CO_2_RR and NRR: (i) targeting molecularly defined active sites, (ii) increasing the local reactant concentration at the active sites, and (iii) stabilizing and confining product intermediates.

## 1 Introduction

**Figure 1:**
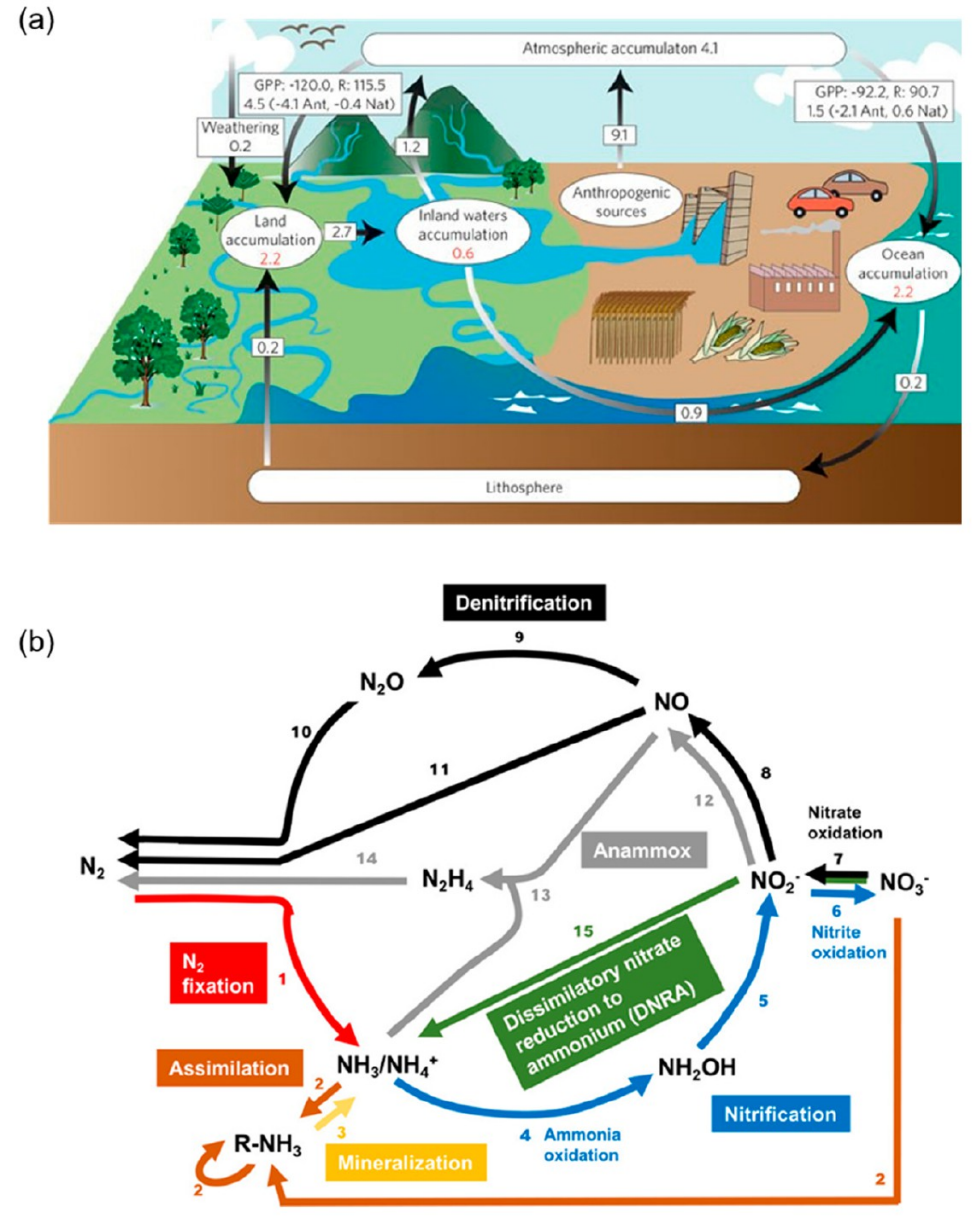
(a) Scheme of the carbon cycle. Reproduced with permission from ref (3). Copyright 2009 Springer Nature. (b) Cycle of biologically driven N transformations that occur in natural and human-influenced terrestrial and marine environments. Reproduced with permission from ref (6). Copyright 2020 American Chemical Society.

Many of today’s environmental, economic, and societal issues are related to the transformation of two inert gases, N_2_ and CO_2_. The transformation of N_2_ via the Haber–Bosch process accounts for over 1% of the world’s energy consumption,^1^ providing nitrogen fertilizers required to sustain the current global food production. Meanwhile, the amount of CO_2_ released into the atmosphere from the combustion of fossil fuels has reached unprecedented levels, further accelerating climate change.^2−6^ Both CO_2_ and nitrogen undergo complex environmental cycles (Figure 1a and b), increasing the challenges associated with their capture and conversion. Implementing sustainable cycles for CO_2_ and N_2_ and minimizing their environmental impact is critical, as recently highlighted in the latest Intergovernmental Panel on Climate Change (IPCC) report or in Europe in the EU green deal and Fit for 55 packages.^7−9^

The electrochemical conversion of CO_2_ and N_2_ into value-added products or net zero commodities such as materials, renewable fuels, and energy vectors appears as an appealing solution in this context, as it can utilize sustainable sources of electricity powered by solar, wind, wave, and hydro energy to promote reactions currently carried out using fossil fuels. This approach would provide a carbon-neutral route to C- and N-containing products while enabling the efficient storage of intermittent renewable sources of electricity as chemical bonds, largely overperforming the battery storage energy efficiency.^10^

The main hurdle to developing energy efficient processes for converting nitrogen to ammonia and carbon dioxide to energy dense products such as hydrocarbons is selectivity. The chemical inertness of these reactants disadvantages their transformation compared to more kinetically facile reactions such as the hydrogen evolution reaction (HER). Furthermore, selectivity is one of the most challenging aspects to address when developing electrocatalysts to mediate the CO_2_ reduction reaction (CO_2_RR) and the nitrogen reduction reaction (NRR). In both cases, multiple reaction products are typically observed. These result from the reduction of CO_2_ and N_2_ themselves as well as from the proton sources used to mediate these reduction reactions, which involve successive coupled electron–proton transfers. In this regard, the electrocatalyst microenvironment plays a vital role and can be engineered to improve selectivity through three key strategies: (i) targeting a narrow distribution of molecularly defined active sites, (ii) increasing the reactant/proton ratio at the three-phase interface where the reaction takes place to lower the undesired formation of H_2_, and (iii) the stabilization and confinement of reaction intermediates in the electrode vicinity to favor the formation of multielectron reduction products. While there exists an extensive amount of literature in both the CO_2_RR and NRR fields, including several recent reviews of specific subtopics,^11−14^ we aim in this Review to illustrate through a handful of selected examples the key strategies for increasing selectivity toward value-added products.

After a brief explanation of the kinetic and thermodynamic origins of multiple product generation in the CO_2_RR and NRR, we discuss the key factors in catalyst design in steering product selectivity, namely nanostructuring, surface functionalization, control of crystal size and facets, and single-site engineering. We then explore the impact of the electrolyte on the activity at the electrode surface, including aspects such as pH, the alkali metal cation, and the use of novel electrolytes. The final section focuses on the implementation and optimization of triple-phase interfaces to improve the local reactant concentration and mass transport. We conclude with our perspectives on this rapidly growing topic and where we envisage future challenges and opportunities to lie.

It is important to note that the NRR field has been strongly affected by a series of false positives, and a standardized set of experiments has been outlined to identify, quantify, and eliminate experimental artifacts.^15^ Ammonia contamination may arise from sources such as the air, chemicals ,and the experimental setup, which is particularly significant when the quantity of ammonia produced in the NRR is very low. Additionally, labile nitrogen-containing compounds such as nitrates, nitrites, nitrogen oxides, and amines are often present in the N_2_ gas stream, the air, and the catalyst itself. To reliably attribute ammonia production to the NRR, quantitative isotope measurements with ^15^N_2_ gas and the removal of impurities from the gas stream are imperative. To preserve a fair comparison of performance between catalytic materials, in this Review we present only examples that follow the guidelines provided in the above ref (14), unless clearly stated otherwise.

## 2 Mechanistic and Thermodynamic Origin of Multiple Product Generation in CO_2_RR and NRR

Both the CO_2_RR and NRR to value-added products involve multiple successive proton-coupled electron transfers (Table 1), which represent a significant kinetic challenge to overcome to achieve high selectivity, particularly compared to the more kinetically facile two-electron hydrogen generation reaction.^16−18^ This kinetic challenge is further complexified by the low availability of the reactants, as both CO_2_ (∼33 mM at P_CO2_ = 1 atm) and N_2_ (∼0.7 mM at P_N2_ = 1 atm) have typically poor solubility in water.^19^ In the context of the CO_2_RR to multicarbon products, the low solubility of the primary reaction products such as CO also decreases the overall catalyst selectivity for multicarbon products, which result from the subsequent reduction of these primary products.

**Table 1:** Selected Standard Potentials of CO_2_ and N_2_ in Aqueous Solutions (V vs SHE) at 1.0 atm and 25 °C Calculated According to the Standard Gibbs Energies of the Reactants in Reactions^a^

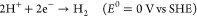 1
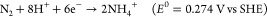 2
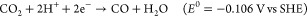 3
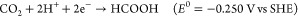 4
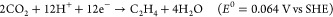 5
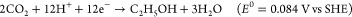 6

a Reproduced with permission from refs (21) and (23). Copyright 2014 Royal Society of Chemistry and 2019 Royal Society of Chemistry, respectively.

**Figure 2:**
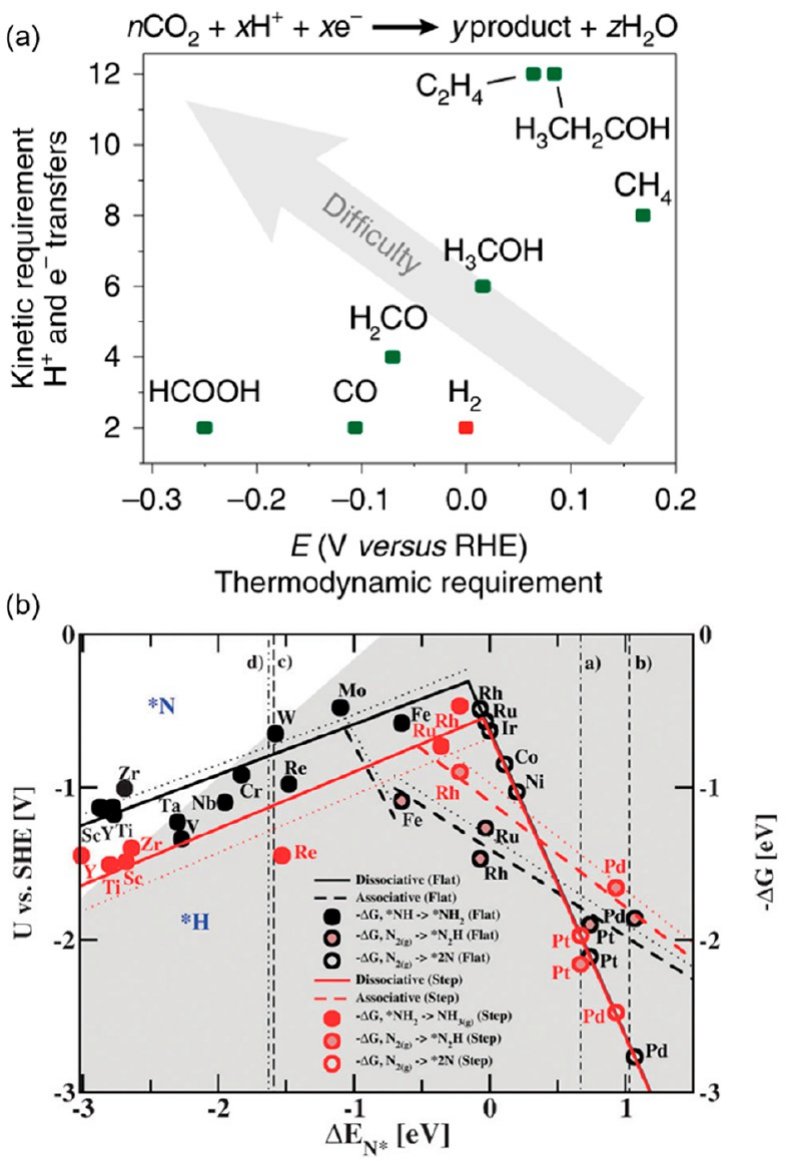
(a) Kinetic versus thermodynamic requirements of various CO_2_ reduction reactions. The plotted values are based on the reaction equation given above the graph, made stoichiometric according to the product composition. Reproduced with permission from ref (22). Copyright 2019 Springer Nature. (b) Combined volcano diagrams (lines) for the flat (black) and stepped (red) transition metal surfaces for the reduction of nitrogen with a Heyrovsky-type reaction without (solid lines) and with (dotted lines) H-bond effects. Reproduced with permission from ref (24). Copyright 2012 Royal Society of Chemistry.

In addition, a thermodynamic challenge is associated with the CO_2_RR, since proton reduction (HER) is more thermodynamically favorable than the reduction of CO_2_ to most products (Figure 2a and eqs 3–6).^20−22^ Although less critical in the case of NRR, the standard electrochemical potential for the proton reduction reaction is yet close to that of the nitrogen reduction reaction (NRR) at 0.057 V vs. SHE (eq 2).^23^ The intrinsic stronger binding of H atoms over N_2_ on most metal surfaces, highlighted in Figure 2b, further illustrates the challenge to increase NRR selectivity vs HER.^24^

This illustrates the three main challenges (thermodynamic, kinetic, or related to the mass transport of the reactants and primary reaction products) that must be overcome to reach high selectivity in the CO_2_RR and NRR. We will review in the next sections the three main axes currently explored toward that goal, focusing on catalyst design, electrolyte engineering, and three-phase interface modulation.

## 3 Increasing Selectivity via Catalyst Design

### 3.1 Catalyst Nanostructuring for Improved Mass Transport

Advancements in nanotechnology and characterization techniques have enabled a plethora of morphologies to be explored to improve catalytic activity and product selectivity. Porous materials have attracted particular attention due to their effect on the local chemical environment, including local pH and the mass transport of the reactant and intermediates.^25,26^ The ability to increase the number of effective active sites, both by maximizing surface area and facilitating the accessibility of such sites, makes porosity useful and interesting across a broad range of fields.^27^ Such effects are especially crucial when considering the poor solubility of CO_2_ and N_2_ in aqueous electrolytes, which causes mass transport limitations and barriers to high activity and selectivity.

Hierarchical porous networks are found commonly in biological organisms as a strategy to mitigate mass transport limitations in the utilization of nutrients.^28^ The three-dimensional networks were replicated in early work by Huan et al., who used gold nanodendrites for electrochemical sensing.^29^ Their application in catalysis has recently appeared as an efficient strategy to increase current densities and catalyst selectivity in small-molecule electroreduction and oxidation.

**Figure 3:**
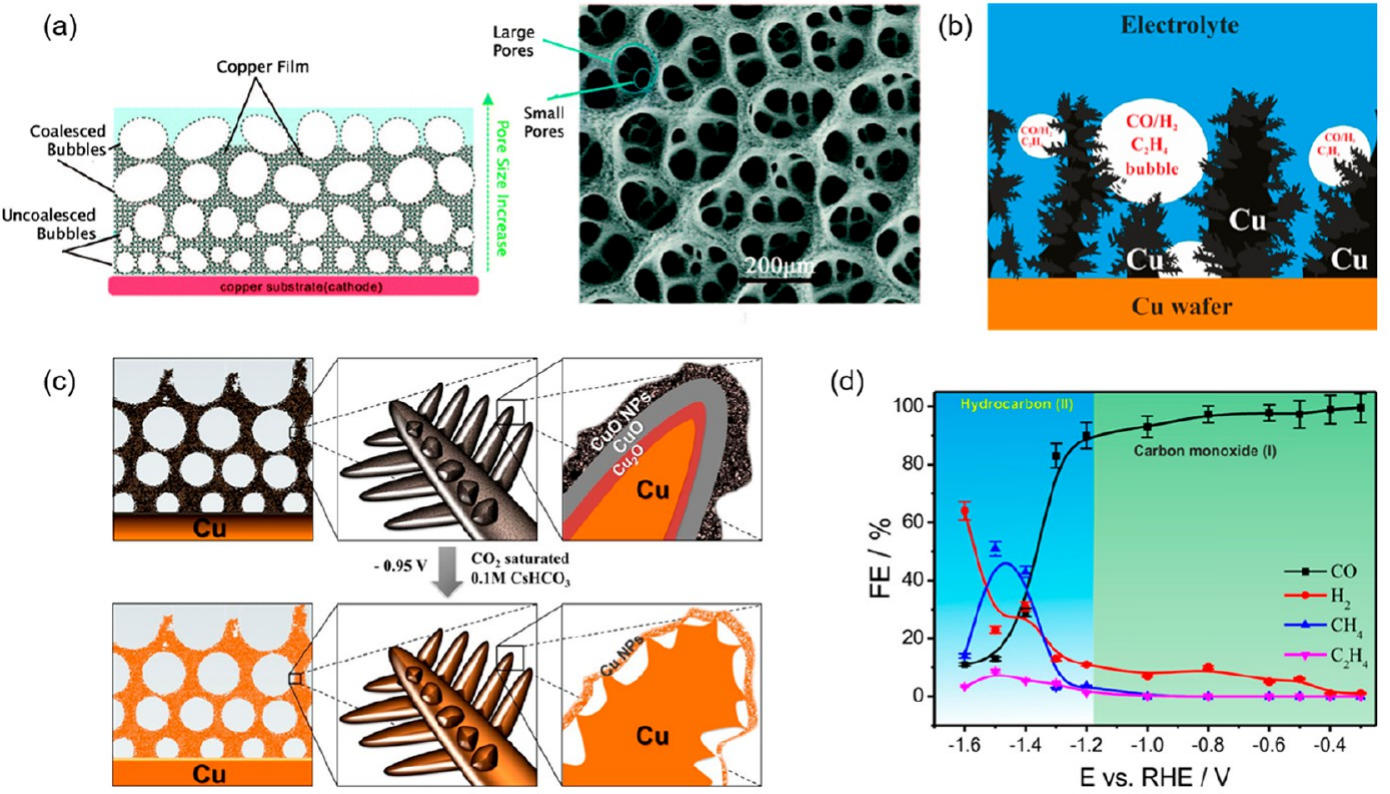
(a) Schematic illustration and SEM image of a copper DHBT foam, demonstrating the hierarchical pore structure. Reproduced with permission from refs (30) and (43). Copyright 2015 Royal Society of Chemistry and 2013 IOP Publishing, respectively. (b) Schematic illustration of gaseous CO_2_R intermediates (CO and C_2_H_4_) and byproducts (H_2_) trapped within the porous Cu foam catalyst. Reproduced with permission from ref (41). Copyright 2016 American Chemical Society. (c) Schematic illustration of a dendritic CuO DHBT foam before (top) and after (bottom) CO_2_ electroreduction in 0.1 M CsHCO_3_, showing the material reduction to metallic Cu and the formation of nano-Kirkendall voids. Reproduced with permission from ref (47). Copyright 2019 Proceedings of the National Academy of Sciences. (d) Potential-dependent product distribution of the CO_2_RR using a Ag-DHBT foam catalyst by Faradaic efficiency, showing the formation of hydrocarbons at potentials more negative than −1.2 V vs RHE. Reproduced with permission from ref (33). Copyright 2018 American Chemical Society.

The dynamic hydrogen bubble templating (DHBT) method has been the most prominent technique to create such hierarchical porosity, which was recently comprehensively reviewed by the Bhargava group^30^ and specifically for CO_2_RR materials by the Broekmann group.^31^ The process involves the electrodeposition of a metal from aqueous solutions of the respective cations, while cogenerated hydrogen bubbles act as a dynamic template to create a metal foam. As the bubbles nucleate, grow, and detach, a hierarchical pore structure forms with layers of pores of increasing diameter (Figure 3a), including micropores in the submicrometer range and macropores in the range of 10–100 μm.^30^ The DHBT technique is relatively simple, requiring aqueous solutions and no need for organic or inorganic templates (as in traditional metal foam synthesis),^32^ high temperatures, high pressures, or uncommon equipment. Nonetheless, additives such as citrate are common to influence crystal growth.^33−35^ The formation of Bi and multimetallic catalysts are also possible by coelectrodeposition, galvanic replacement, stepwise electrodeposition, or spontaneous decoration.^30^ For example, many studies for CO_2_RR have coupled copper with one other metal such as Ag, Sn, In, or Zn.^36−40^

By fine-tuning parameters such as the proton source and concentration, the applied overpotential or current density, the substrate material, and the metal source and concentration, the nanostructure can be carefully controlled and optimized. Broekmann and co-workers produced a dendritic Cu-based DHBT foam and demonstrated a strong dependence of the C_2_-product selectivity on the surface pore size diameter, with the optimal size being between 50 and 100 μm.^41^ They identified the temporal trapping of gaseous intermediates inside these pores as the key to product selectivity. Intermediates such as CO and C_2_H_4_, which would otherwise be released into the bulk electrolyte, were entrapped in the pores of the foam catalyst, causing them to further react to form C_2_H_6_ (Figure 3b). At −0.8 V vs RHE, the authors achieved a 55% Faradaic efficiency for C_2-_products.

Such dendritic structures with large surface areas are common in this synthesis due to the deposition taking place at high current densities and therefore in the diffusion-limited regime. Copper- and oxide-derived copper dendrites have attracted particular interest due to their apparent selectivity for multicarbon products.^42−45^ Huan et al. produced a dendritic CuO material from DHBT that could be used as both CO_2_R and OER catalysts.^46,47^ It consisted of a triple-layer structure with a metallic Cu core covered by layers of Cu_2_O and CuO (Figure 3c). In electrocatalytic conditions, the CuO material is reduced to metallic Cu, generating nano-Kirkendall voids within the dendrite structures. Such voids, which appear at the copper–copper oxide interface upon reduction, are termed nano-Kirkendall voids, as they appear as a consequence of the very different diffusivities of Cu and O atoms. The overall external shape of the material is maintained upon reduction, but cavities are generated under its external layer due to the lower density of Cu with respect to the original copper oxides.^48^ These gas-accessible voids were proposed to enhance the confinement of secondary CO_2_RR products, such as CO, resulting in FE_C2+_ over 50%. By using the catalyst in a continuous flow electrolyzer, they were able to reach a stable current of 25 mA/cm^2^ with 2.95 V, equating to 21% energy efficiency for hydrocarbon production. By coupling the cell to a photovoltaic cell, they achieved a 2.3% solar-to-hydrocarbon efficiency.

DHBT foams for single-carbon products such as CO and formate have also been reported. A silver foam with needle-shaped features in the mesopores was produced by using a citrate additive to control growth on the nanometer scale.^33^ Between −0.3 to −1.2 V vs RHE, 90% Faradaic efficiency for CO was observed; however, at higher overpotentials the foam produced C_2_ products, with 51% CH_4_ at −1.5 V (Figure 3d). This unusual activity for Ag was attributed to the catalyst morphology and nanostructure increasing the *CO surface concentration and residence time. Recent work by Mayer and co-workers exemplifies the advantages of the simplicity of the DHBT method. In a one-step synthesis they used waste industrial Cu–Sn bronze as a material precursor to deposit a mesoporous Cu_10_Sn foam.^49^ They achieved over 85% Faradaic efficiency for CO at −0.8 V vs RHE, over double that of the plain Cu–Sn bronze, with partial current densities three times higher. Du et al. prepared a nanoporous tin DHBT foam on a tin substrate and achieved a Faradaic efficiency for formate of 90% with current densities of 23 mA/cm^2^.^50^

**Figure 4:**
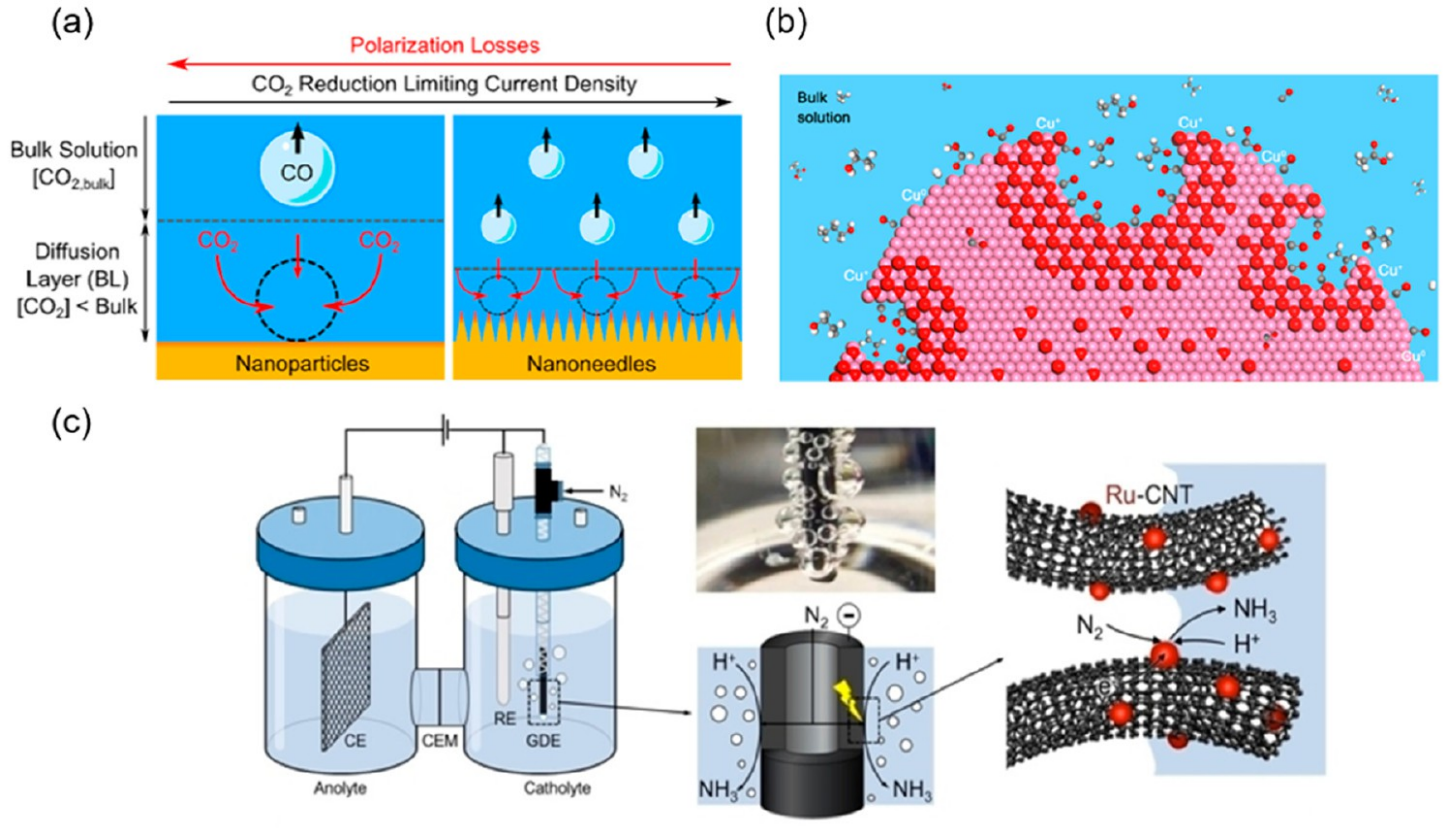
(a) Schematic showing the effect of the catalyst nanostructure on the bubble departure diameter and its impact on the diffusion boundary layer thickness and CO_2_ mass transport. Reproduced with permission from ref (51). Copyright 2017 American Chemical Society. (b) Schematic of a cuprous oxide catalyst with nanocavities that confine carbon intermediates such as CO and C_2_H_4_. Color code: white, hydrogen; gray, carbon; red, oxygen; and pink, copper. Reproduced with permission from ref (53). Copyright 2020 American Chemical Society. (c) Schematic illustration (left to right) and picture (middle-top) of the NRR in an H-cell with a microtubular Ru-CNT (carbon nanotube) gas diffusion electrode. Reproduced with permission from ref (55). Copyright 2020 Wiley.

Other morphology-based strategies have been utilized to modulate mass transport in CO_2_ reduction, including the application of nanostructures such as nanowires, sheets, needles, cones, or tubes. Burdyny et al. explored the effect of the nanomorphology of a silver catalyst on gas evolution and subsequently bubble-induced mass transport.^51^ By combing mathematical modeling and experimental observations using a dark-field microscope, they compared bubble formation on nanoparticles, nanorods, and nanoneedles and found mean bubble diameters of 97, 31, and 23 μm, respectively. They illustrated that the generation of smaller bubbles improved long-range mass transport of CO_2_, resulting in a small diffusion thickness and a fourfold increase in the limiting current density of CO production (Figure 4a). Surendranath and co-workers synthesized gold inverse opal thin films and found that changing the mesostructure by increasing the porous film thickness could diminish HER 10-fold while maintaining activity for CO_2_ to CO, increasing the faradaic efficiency for CO from less than 5% to over 80%.^52^ They attributed this to the formation of diffusional gradients. Studies of nanocavities and their performance and mechanism of action have emerged in recent years. Yang et al. utilized finite-element method simulations and experimental measurements on a multihollow cuprous oxide catalyst.^53^ Analysis from X-ray absorption studies and operando Raman spectra indicated that the pore cavities confined *CO intermediates, which bound to Cu^+^ sites and locally protected them against reduction during CO_2_RR (Figure 4b), as well as promoted C–C coupling. The authors achieved a C_2+_ product Faradaic efficiency of 75% and a partial current density of 267 mA/cm^2^.

As N_2_ electroreduction is a comparatively less mature field with its own unique challenges, studies into morphological effects on catalytic activity and selectivity are less extensive. Although a range of nanostructures exist among the literature,^54^ specific insight into the role morphology plays in catalysis is limited. Wei et al. loaded ruthenium nanoparticles onto carbon nanotubes, which were also applied as the gas diffusion electrode.^55^ Despite using a typical H-cell setup, the GDE structure allowed N_2_ gas to flow through the GDE and porous catalyst instead of being solely solubilized in the electrolyte, as illustrated in Figure 4c. They achieved a NH_3_ yield rate of 2.1 nmol/cm^2^ · and Faradaic efficiency of 13.5%.

A great range of nanostructures have been applied to the CO_2_RR and NRR to regulate mass transport, and although strong correlations between structure and performance have been made, their mechanisms of action are often highly complex and difficult to define. Most theories focus on the mass transport of reactants and intermediates either through improved diffusion and convection or through their physical confinement in the catalyst pores. Considerable progress has been made by combining computational and experimental research to define and improve catalyst nanomorphology, especially in the CO_2_RR field; however, their application to new materials and fields such as NRR is still an open area of research.

### 3.2 Surface Functionalization

Functionalization of the electrode or catalyst surface with organic or inorganic ligands has been explored as a strategy to tune the interaction between adsorbed intermediates and catalysts, inhibiting HER and improving product selectivity. In addition to the decoration of the surface of a catalytic material with surface-bound ligands, the covalent grafting of molecular cocatalysts onto the surface of a catalytic material has also been explored as a strategy to further tune the catalyst selectivity.^56^

**Figure 5:**
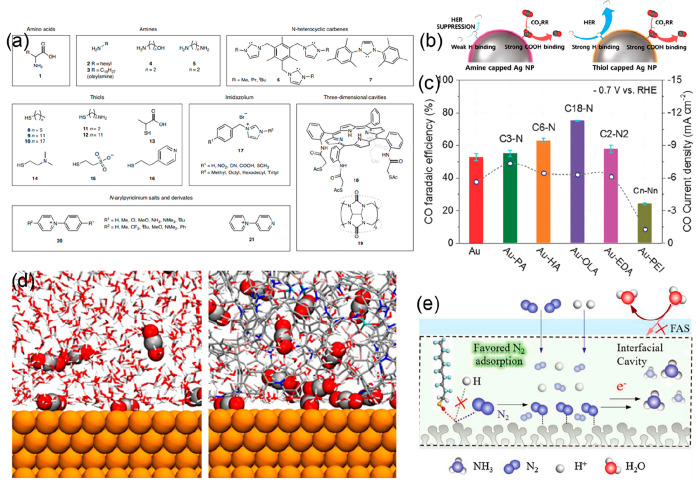
(a) Surface modifiers grouped into different classes used to modulate the local chemical environment around the catalytic site (amino acids, amines, *N*-heterocyclic carbenes, thiols, imidazolium, three-dimensional cavities, *N*-arylpyridinium salts, and derivatives). Reproduced with permission from ref (26). Copyright 2020 Springer Nature. (b) Schematic of the product selectivity depending on the Ag NPs immobilized with an amine (or thiol)-containing anchoring agent. Reproduced with permission from ref (58). Copyright 2017 American Chemical Society. (c) FE_CO_ (column) and *j*_CO_ (circle) of gold catalysts with different surface amine modifications in CO_2_-saturated 0.1 M KHCO_3_ at −0.7 V vs. RHE. Reproduced with permission from ref (59). Copyright 2018 Wiley. (d) Interface structure after 12 ns molecular dynamics simulations with a water–Cu interface and a random copolymer with a water–Cu interface. Color code: Cu, orange; C, gray; O, red; N, blue; F, pink; S, cyan; and H, white. Reproduced with permission from ref (68). Copyright 2021 American Chemical Society. (e) Possible NRR mechanism at the surface of the hydrophobic catalyst. Reproduced with permission from ref (73). Copyright 2021 Elsevier.

In this section, we will outline some key examples in the diverse field of catalyst surface functionalization, which has been comprehensively reviewed for the CO_2_RR by Reisner and co-workers.^26^ To date, many organic additives such as amino acids,^57^ amines,^58,59^ aminothiols,^60^ pyridiniums,^61,62^*N*-heterocyclic carbenes (NHCs),^63,64^ imidazolium ligands,^65^ porphyrin-based metallic complexes,^66,67^ polymers,^68,69^ and inorganic additives,^70,71^ have been proposed to control the binding energies of CO_2_RR reaction intermediates (Figure 5a). For instance, Kim et al. demonstrated a 94.2% FE for the production of CO from amine-capped Ag supported on carbon, thanks to the effective suppression of the HER and the intrinsic high selectivity toward the CO_2_RR from Ag (Figure 5b).^58^ DFT calculations suggested that the amine-capped Ag nanoparticles stabilize the *COOH intermediate while destabilizing *H. Conversely, thiol-capped Ag nanoparticles exhibited superior reaction rates toward both the HER and CO_2_ reduction by indiscriminately increasing ΔG_*H_ and ΔG_*COOH_.

As presented in Figure 5c, Zhao et al. developed a simple modification strategy using amines to depress the hydrogen evolution reaction on ultrasmall Au NPs and enhance CO_2_-to-CO conversion.^59^ The amine groups, as well as the molecular configuration, were found to play important roles in tuning the electrocatalytic activity of low-coordinated sites of the nanoparticles. The authors claimed that strong interactions between the Au surface and the amine ligands combined with the peculiar configuration were responsible for the improved CO_2_RR performance. Remarkably, linear amines promoted the formation of CO, an effect that was enhanced by increasing the length of the alkyl chain, whereas the branched polyamine greatly depressed it. Wang et al. demonstrated 55% and 77% selectivities for ethylene and C_2+_ products, respectively, using a tricomponent copolymer to modify the surface of Cu electrodes (Figure 5d).^68^ Systematic studies indicated that the three components of the copolymer control electrostatic interactions, gas diffusion, and hydrophilicity, which were found to be necessary to improve selectivity. The copolymer was obtained by ring-opening metathesis polymerization, thereby offering a new degree of freedom for tuning the selectivity.

Applying a molecular design approach to tune heterogeneous catalysts has also proved effective in the functionalization of palladium foil with chelating *N*-heterocyclic carbene (NHC) ligands, demonstrating a 32-fold increase in activity for CO_2_ to C_1_ products.^63^*N*-Aryl-pyridinium salts have also proved effective in tuning electronic properties to stabilize intermediates for CO_2_RR to ethylene.^62^ Porphyrin-based metallic complexes have been used to functionalize copper surfaces to increase the concentration of CO intermediates and promote C–C coupling; a Faradaic efficiency of 41% for ethanol was achieved at 124 mA/cm^2^ at −0.82 V vs RHE.^67^

Modifying the catalyst surface indirectly has also been implemented by Varela et al. through the addition of halides to the electrolyte.^71^ They hypothesized that the adsorption of halides onto copper increased the negative charge of the catalyst surface, altering the selectivity. In the case of iodide, the induced negative charge favored the protonation of CO, enhancing CH_4_ production.

Applying well-defined molecular approaches to heterogeneous systems can give important insights into catalytic mechanisms and help to fine-tune active sites and product selectivity. Some functionalization strategies operate through molecular coordination and can therefore be carefully controlled by altering functional and side groups so that specific CO_2_RR intermediates can be stabilized. Other strategies, such as the addition of halides or ionic liquids, affect the charge on the catalyst surface, increasing CO_ads_ coverage for example.^70,72^ Both have proven effective in improving product selectivity in the CO_2_RR, and similar approaches could be applied to the NRR to help overcome the dominance of HER (Figure 5e).^73^ The exact surface binding motifs of ligands and the mechanism for altered selectivity are still unclear. Understanding the precise nature of the interface remains a key challenge for attaining the desired catalytic properties.^56^

### 3.3 Crystal Size and Facet Control

**Figure 6:**
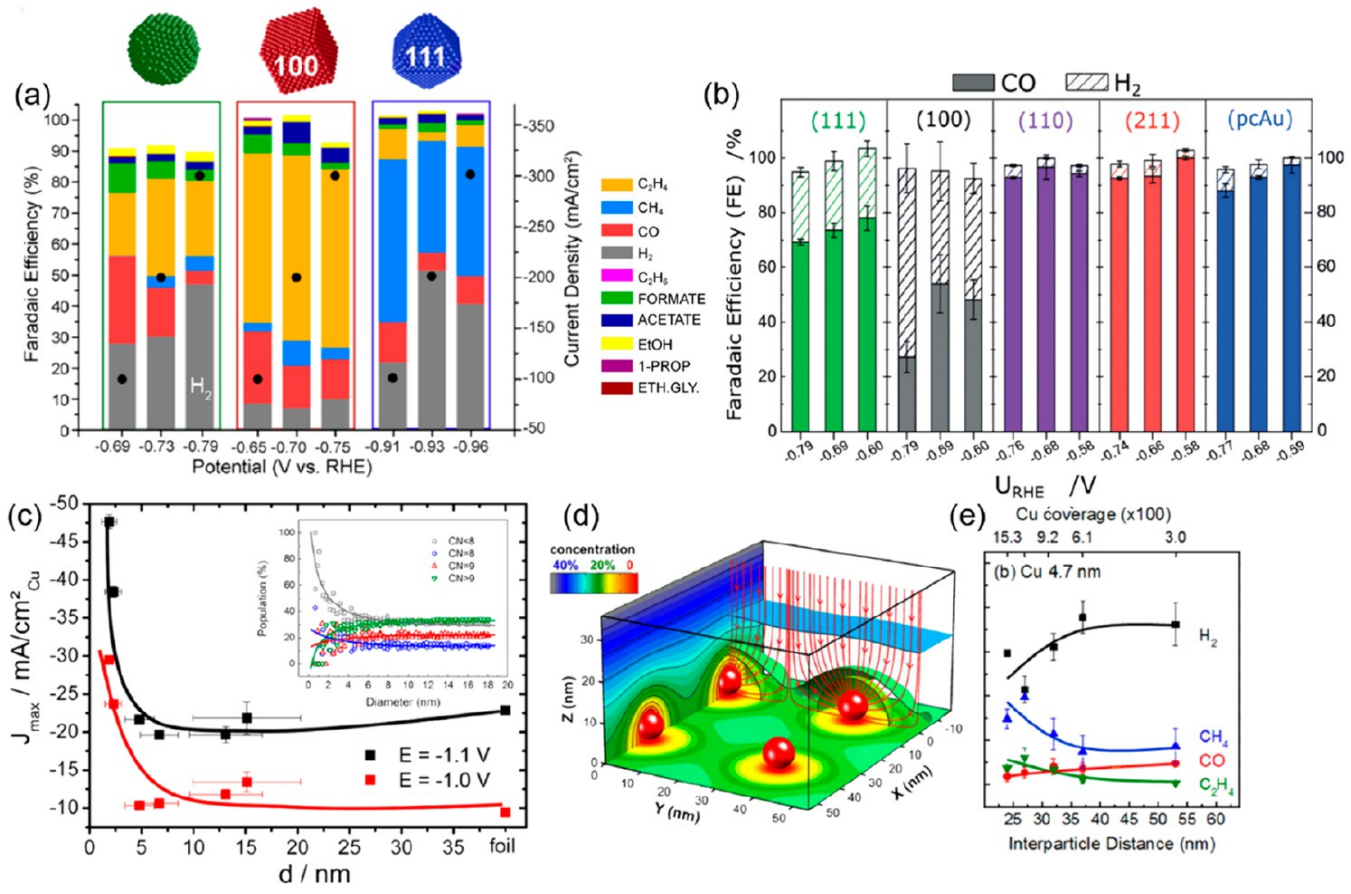
(a) Relation between the Faradaic efficiencies and potentials for different Cu morphologies (sphere, cube, and octahedra). Reproduced with permission from ref (74). Copyright 2020 American Chemical Society. (b) Relation between the Faradaic efficiencies and potentials with the exposure of different Au facets. Reproduced with permission from ref (75). Copyright 2019 Wiley. (c) Particle size effect during catalytic CO_2_ electroreduction. The Faradaic current densities at −1.1 and −1.0 V vs. RHE are plotted against the size of the Cu NP catalysts, and the inset shows the population (relative ratio) of surface atoms with a specific coordination number (CN) as a function of the particle diameter. Reproduced with permission from ref (76). Copyright 2014 American Chemical Society. (d) Simulation results of the CO_2_ concentration distribution based on diffusion equations. The red arrows show the reactant flux toward the NPs. The color scale shows the concentration of CO_2_ at a given distance from the NPs as a percentage of its value in the bulk of the electrolyte. A diffusion layer thickness of 100 nm was assumed. Reproduced with permission from ref (77). Copyright 2016 American Chemical Society. (e) Faradaic selectivity during the electroreduction of CO_2_ at −1.1 V vs. RHE with a Cu interparticle distance of 4.7 nm. Reproduced with permission from ref (79). Copyright 2016 American Chemical Society.

Tremendous advances have recently been made to engineer catalysts in order to limit the HER during the CO_2_RR and NRR processes.^72^ Compared with their bulk counterparts, nanostructured catalysts show original and often enhanced activities due to their unique surface electronic and chemical properties. These properties can be finely adjusted to tune the activity and selectivity of electrocatalytic reactions. The surface of a nanomaterial catalyst typically consists of planar areas with single-crystalline orientations separated by steps and kink sites with lower coordination numbers. Complex atomic structures are therefore present at the interface between different grains in polycrystalline and/or nanostructured surfaces. Buonsanti et al. investigated the catalytic properties of exposed facets of Cu nanocatalysts at commercially relevant current densities (Figure 6a).^74^ The study revealed that facet-dependent selectivity is retained in a gas-fed flow cell, showing greater HER suppression than in a conventional H-cell. The (100) facets of Cu nanocubes have been identified to be selective for the evolution of C_2_H_4_, whereas the (111) facets of Cu octahedra are selective toward CH_4_. Conversely, Cu spheres do not exhibit any specific product selectivity, suggesting that randomly mixed facets cannot depress the HER during the CO_2_RR. Chorkendorff et al. systematically investigated the structure–selectivity relationship of Au single crystals for electrocatalytic CO_2_ reduction (Figure 6b).^75^ Remarkably, they found that the kinetics for the formation of CO strongly depend on the surface structure. Under-coordinated sites, for instance, those on the surface of Au(110) or at the step edges of Au(211), show at least 20-fold higher activities than more coordinated configurations, such as Au(100). By selectively poisoning under-coordinated sites with Pb, the authors identified the selectivity of these active sites toward the reduction of CO_2_, effectively suppressing the HER.

Roldan Cuenya, Strasser, and co-workers investigated the role of particle size in CO_2_ electroreduction using size-controlled Cu nanoparticles (NPs).^76^ A dramatic increase in the catalytic activity and selectivity of CO against H_2_ was observed once the particle size was decreased, particularly for NPs smaller than 5 nm, as shown in Figure 6c. Changes in the population of low-coordinated surface sites and their stronger chemisorption were linked to H_2_ and CO selectivity. As shown in the inset of Figure 6c, a drastic increase in the number of undercoordinated atoms is observed below a particle size of 2 nm, with a coordination number lower than 8. These peculiar sites accelerate both hydrogen evolution and CO_2_ reduction to CO via an increase in binding energy. However, the undercoordinated sites are unfavorable for the subsequent hydrogenation of CO, which lowers the hydrocarbon selectivity of the NPs. A plausible explanation for the observed trend is the reduced mobility of intermediate reaction species (CO and H) on the small NPs due to stronger bonding, which decreases the possibility of further recombination to form hydrocarbons. At intermediate particle sizes, the spherical particle model predicts low and constant populations of (100) and (111) facets, which is consistent with the reduced yet constant hydrocarbon selectivities observed for Cu NPs between 5 and 15 nm compared to Cu bulk surfaces. For these larger NPs, weaker binding of CO and H is expected, favoring hydrocarbon formation.

Another critical parameter for suppressing the HER with metal NP catalysts is the interparticle spacing. Mesoscale phenomena, such as interparticle reactant diffusion and readsorption of intermediates, can play an important role in the product selectivity for multistep reactions.^77,78^ In this context, Mistry et al. showed that, for CO_2_ electroreduction, decreasing the interparticle spacing for a constant nanoparticle size can suppress the HER, which further increases the selectivity for CH_4_ and C_2_H_4_ due to the increased possibility of the *CO intermediate readsorbing on a neighboring particle and being further reduced (Figure 6d and e).^79^ More importantly, this study uncovers general principles of tailoring NP activity and selectivity by carefully engineering the size and distance. These principles guide the rational design of mesoscopic catalyst architectures to enhance the production of the desired reaction products.^80^

### 3.4 Single-Site Engineering

One of the main hurdles to the rational improvement of selectivity using metallic or metal oxide/sulfide catalysts is the large distribution of accessible sites that may result in different favored reaction products and decreased selectivities. Single-atom catalysts (SACs) hence represent an attractive strategy to increase selectivity via a narrower distribution of active sites and improved control of the first coordination sphere of the active site, bridging the gap between well-defined molecular catalysts and complex heterogeneous materials. The catalytic properties of SACs hence result from the combination between the molecular tuning of the coordination environment of the active sites and its interaction with the support.^81^ Different types of supports for SACs have been explored to date and include metals, carbon-based materials, and metal (hydr)oxides, nitrides, and carbides. Metal-supported SACs, also called single-atom alloys (SAAs) have also been explored. They generally yield thermodynamically more stable interactions than other atom-supported interactions due to strong metal–metal interactions.^82^ Advantageously, SAAs can offer different active sites on the host metal (i.e., the support) and the individual atoms, providing further opportunities to modulate reaction pathways.^83−85^ Zhang et al. demonstrated the control of the CO_2_RR products between formate and CO by varying the Cu/Sn composition.^86^ They reported that the use of Cu_1_Sn_1_ comprising a core–shell structure doped with a small amount of Cu using CuSn and SnO alloys as the core and shell, respectively, leads to the preferential formation of formate with an FE greater than 95% at −1.2 V. In contrast, single atoms of Sn supported on Cu:Cu_20_Sn_1_ show a high selectivity for CO with a maximum FE_CO_ of 95.3% at −1.0 V.

**Figure 7:**
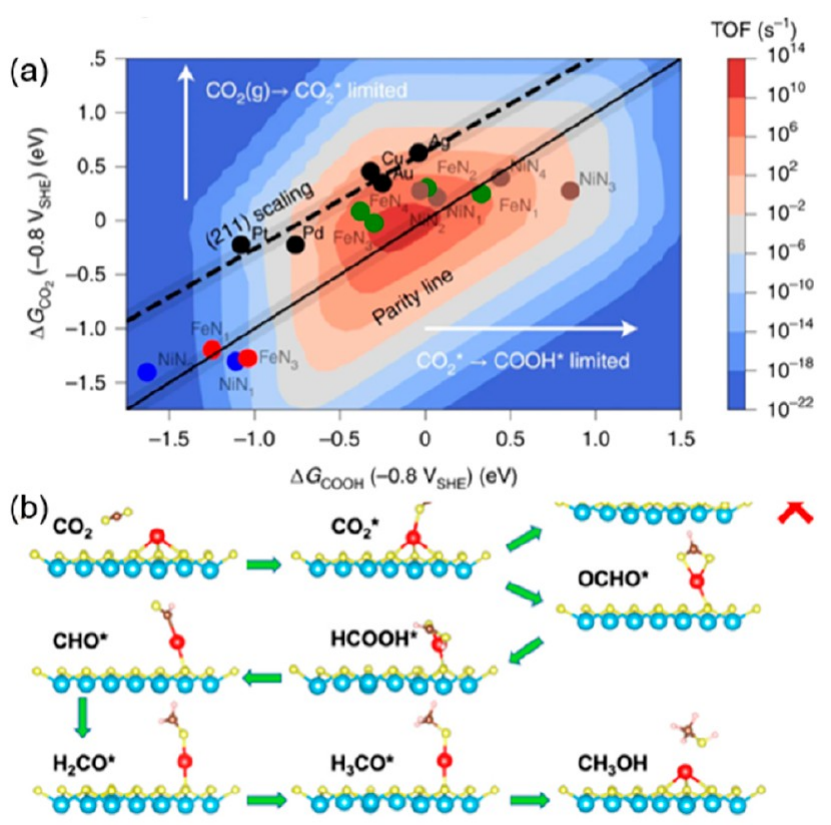
(a) Rate map for the conversion of CO_2_R to CO at −0.8 V_SHE_ and pH = 2 obtained from the (211) TM scaling line. The annotated points show MNC SACs at either single or double vacancies. Reproduced with permission from ref (89). Copyright 2021 Springer Nature. (b) DFT calculation-proposed reaction pathway for the functionalization of CO_2_ to methanol on isolated Cu of SA-Cu-MXene. Reproduced with permission from ref (96). Copyright 2021 American Chemical Society.

Carbon substrates have been widely explored in the form of graphite, graphdiyne, and graphene and its derivatives, including heteroatom (N, O, S, and P)-doped sp^2^ carbon materials. Carbon supports indeed offer several advantages such as high surface area, high electronic conductivity, and strong thermal stability, and they also possess numerous coordination environments to stabilize the single atom sites.^87,88^ The different behaviors of transition metals in the form of nanoclusters or metal–nitrogen-doped carbon catalysts (MNCs) were examined by the Chan and Strasser group.^89^ The results of their calculations revealed that *CO_2_ adsorption is the limiting step on metals, whereas for nitrogen-coordinated SACs the reaction can be limited either by *CO_2_ adsorption or by the formation of *COOH via a proton–electron transfer (Figure 7a). Pan and coauthors reported the design of MNC SACs with atomically dispersed Co sites anchored on polymer-derived hollow N-doped porous carbon spheres.^90^ The single-atom Co–N_5_ sites were identified as the main active centers for CO_2_ activation, and the rapid formation of *COOH as a critical reaction intermediate was followed by a rapid desorption of CO. A similar behavior has also been reported on carbon nanosheet-supported Ni–N_4_ sites, which resulted in near-utility selectivity for CO and a single-pass conversion of 2.6% cm^–2^ when implemented in a flow cell.^91^ Huan et al. investigated a series of iron-based catalysts synthesized by pyrolysis of Fe-, N-, and C-containing precursors for the electroreduction of CO_2_ to CO in an aqueous medium and demonstrated that the selectivity of these materials for CO_2_ reduction is governed by the proportion of isolated FeN_4_ sites compared to Fe-based nanoparticles.^92^ They demonstrated that the nature of the metal species modulates the selectivity of the reaction pathways and suggested that FeN_4_ sites are responsible for CO_2_RR, whereas the Fe cluster are responsible for HER. In a following work, they demonstrated the strong influence of the electrode support on the catalyst selectivity, highlighting the importance of reducing mass transport limitation to promote a higher selectivity toward CO_2_ reduction.^93^

In recent years, metal (hydr)oxides, nitrides, carbides, and sulfides have become very popular supports of SACs thanks to their high specific surface areas, abundant vacancies, and surface functional groups.^94^ Thanks to their strong corrosion resistance, metal nitrides/carbides with metal centers exposed on their surface are good supports to stabilize isolated metal atoms via strong metal–support interactions. In this context, electrically conducting MXenes such as Mo_2_C have been explored as supports for the CO_2_RR.^95^ Zhang et al. demonstrated an efficient approach to produce single atom copper immobilized on MXene for the electrosynthesis of methanol from CO_2_. The SACs were obtained via selective etching of hybrid *A* layers (Al and Cu) in quaternary *MAX* phases (Ti_3_(Al_1–*x*_Cu_*x*_)C_2_).^96^ Combining X-ray absorption spectroscopy analysis and density functional theory calculations, they proposed that the Cu single atoms in the form of Cu^δ+^ with 0 < δ < 2 have a low energy barrier for the rate-determining step corresponding to the conversion of HCOOH* to CHO*, a key reaction intermediate for the reduction of CO_2_ to CH_3_OH (Figure 7b).

## 4 The Electrolyte: An Active Component to Drive Reactivity and Enhance Selectivity

### 4.1 Adjusting the Local pH at the Electrode–Electrolyte Interface

**Figure 8:**
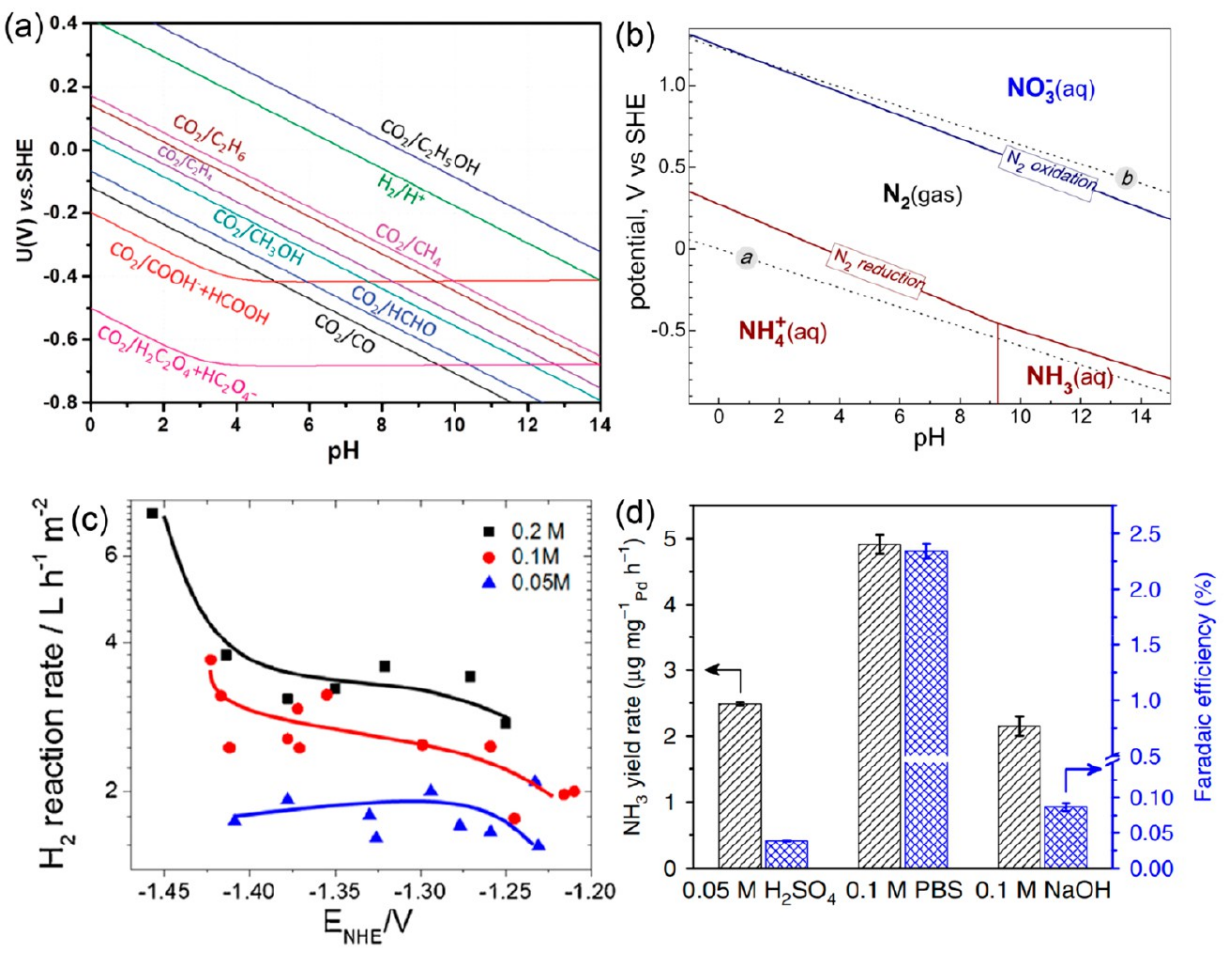
(a) Partial Pourbaix diagram for CO_2_ reduction in aqueous solutions that describes the relationship between the equilibrium potential of the associated reaction and the pH, which is plotted based on thermodynamic data. Reproduced with permission from ref (97). Copyright 2021 Royal Society of Chemistry. (b) Partial Pourbaix diagram for the N_2_–H_2_O system. Solid lines correspond to N_2_ reduction to NH_4_^+^ or NH_3_ (red) and N_2_ oxidation to NO_3_^–^ (blue). Dotted lines *a* and *b* straddle the regions of water reduction to H_2_ and oxidation to O_2_, respectively. Reproduced with permission from ref (98). Copyright 2018 AAAS. (c) Formation rates of gas products as a function of applied electrode potentials in CO_2_-saturated electrolytes with different buffer capacities. Reproduced with permission from ref (106). Copyright 2016 Elsevier. (d) NH_3_ yield rate and Faradaic efficiency of Pd/C processed in N_2_-saturated electrolytes with different pH values. Reproduced with permission from ref (111). Copyright 2018 Springer Nature.

The pH value of the electrolyte greatly influences the equilibrium potential of the CO_2_RR and NRR, as highlighted in the partial Pourbaix diagrams for the CO_2_RR and NRR provided in Figure 8a and b, respectively.^97−100^ A high local pH typically disfavor the HER, thus enabling higher Faradaic efficiencies for multicarbon products in the context of the CO_2_RR and for ammonia in the context of the NRR.^101,102^ The groups of Sinton and Sargent have achieved remarkable results for the CO_2_RR in highly alkaline media; using 7 M KOH, they achieved a 1.3 A/cm^2^ partial current density for ethylene in a flow cell.^103^ Engineering of the triple-phase interface was key to these results and will be discussed further in section 5. Unfortunately for CO_2_ electrolysis, the use of an alkaline electrolyte is complicated by the fatal exergonic formation of carbonate (CO_2_ + 2OH^–^ → CO_3_^2–^ + H_2_*O*/ CO_2_ + OH^–^ → HCO_3_^–^), which is detrimental to both energy and carbon efficiency.^104^ Neutral bicarbonate electrolytes have been applied to reduce electrolyte consumption and to buffer the local pH, although at high currents CO_3_^2–^ is still formed from CO_2_ and electrogenerated OH^–^. Several studies have explored the dependence of the product distribution on the local pH at the electrode–electrolyte interface, as well as the concentration and buffer capability of the electrolyte. In that line, a fine-tuning of the product selectivity for CO_2_RR on Cu electrodes was achieved via the modulation of the local pH upon the variation of the electrolyte buffer capacity, CO_2_ pressure, and current density.^105^ Varela et al. proposed that electrolytes with a high buffer capacity could facilitate the transfer of coupled electrons/protons, thus being beneficial for the evolution of hydrogen.^106^ By comparison, they found that electrolytes with a low buffer capacity could suppress the formation of H_2_ due to the low concentration of protons near the electrode surface, favoring selectivity toward the formation of C_2_H_4_ (Figure 8c). Conversely, applying a higher current density can also lead to a higher local pH. This is due to a high consumption rate of local protons compared to the rate of mass transport of protons from the bulk electrolyte. Huang et al. modeled an electrode surface and found that, even in highly acidic electrolytes (pH = 1), local neutrality and alkalinity could be created above 200 mA/cm^2^.^107^ They required at least 400 mA/cm^2^ to produce multicarbon products. This improved the carbon efficiency considerably, although energy efficiency remains problematic. While a higher CO_2_ pressure could result in a lower local pH at a constant electrolyte concentration, they demonstrated that it also favored ethylene formation by increasing the local *CO concentration and the corresponding *CO surface coverage.^108^ Recently, Chen et al. reported that adjusting the thickness of a highly porous Au film allows the control of the mass transfer resistance and increases the local pH at the electrolyte–electrode interface of CO_2_ reduction, which results in the promotion of the CO_2_RR while inhibiting the HER.^109^

For the nitrogen reduction reaction, Xu et al. summarized the dependence of the formation of nitrogen-reduction intermediates on pH for aqueous media.^110^ Due to the large overpotentials needed to activate N_2_ and the low solubility of N_2_ in aqueous electrolytes, when the applied overpotential is sufficient to trigger the electrochemical synthesis of NH_3_, the reaction at the active sites quickly becomes controlled by the mass transport of N_2_ molecules. Consequently, the presence of protons near the electrode surface leads to the undesired production of hydrogen. As illustrated in Figure 8d, Wang et al. gauged the NRR performance of commercial Pd/C in electrolytes with different pH values. Their observations revealed that the effective suppression of the HER activity in the neutral electrolyte was attributed to a higher barrier for mass and charge transfer.^111,112^

### 4.2 Optimizing the Components of the Electrolyte: Alkali Metal Cation Effects

**Figure 9:**
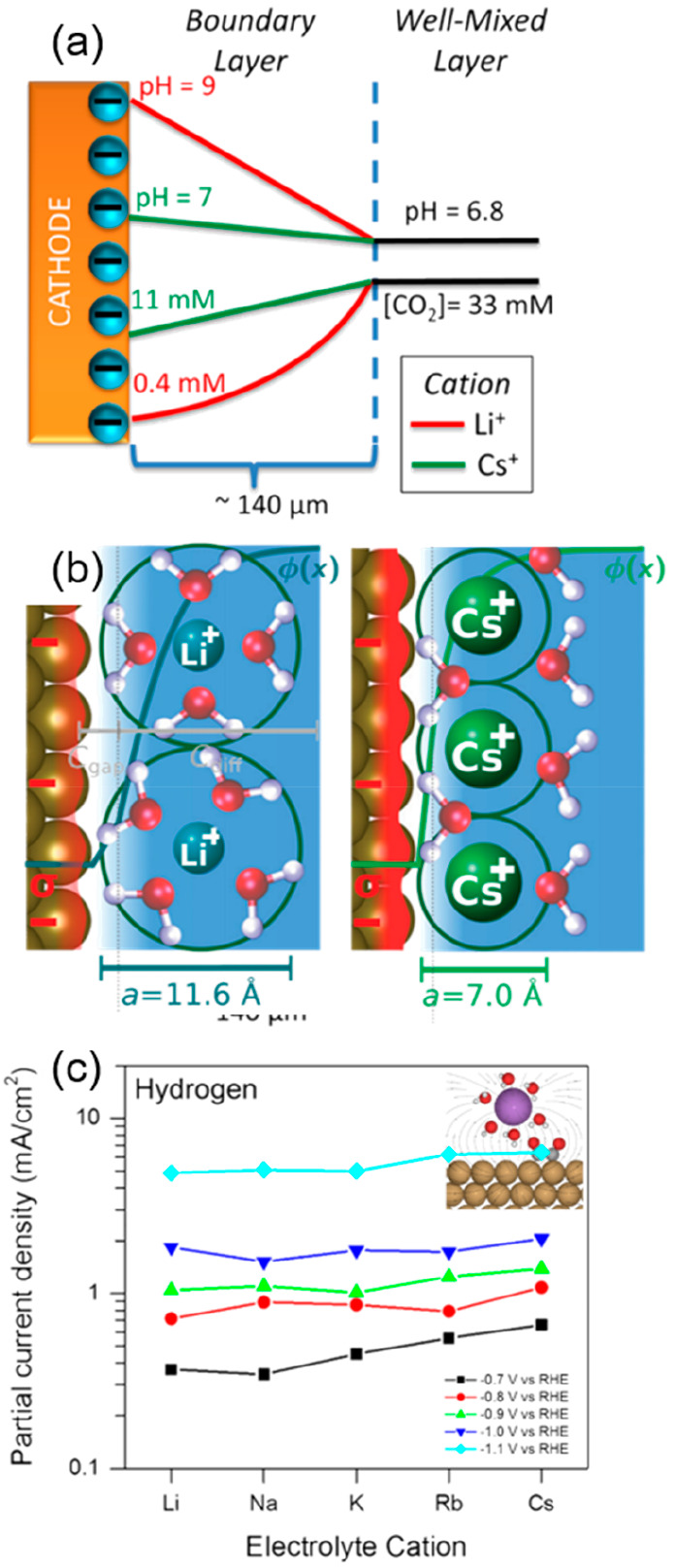
(a) Effect of cation hydrolysis on the electrochemical reduction of CO_2_ over Ag. Distribution of pH and the CO_2_ concentration in the boundary layer. Hydrated Cs^+^ buffers the cathode to maintain the pH close to 7 and increase the CO_2_ concentration. Reproduced with permission from ref (119). Copyright 2016 American Chemical Society. (b) Illustration of the origin of cation effects in field-driven electrocatalysis. Repulsive interactions between hydrated cations at the outer Helmholtz plane reduce the local concentration of cations, the surface charge density (depicted by the red-colored region), and the electric double layer field. The diffuse layer that is explicitly modeled by the size-modified Poisson–Boltzmann (MPB) model is depicted, as well as the Helmholtz gap capacitance region and the interfacial ion diameter. Reproduced with permission from ref (120). Copyright 2019 Royal Society of Chemistry. (c) Average current densities obtained during bulk electrolysis as a function of metal cations at different potentials. Reproduced with permission from ref (122). Copyright 2017 American Chemical Society.

Bicarbonate or carbonate are the most investigated electrolyte salts employed for the CO_2_RR, as they provide a near-neutral pH but most importantly allow a stable and high dissolved CO_2_ concentration to be maintained upon operation.^113,114^ Hence, while the nature of the anions are rarely explored in electrochemical studies, a wide range of studies have investigated the variation of the alkali cations. In the CO_2_RR, while the influence of alkali cations on product selectivity and catalyst efficiency are commonly accepted,^74,115,116^ the origin of this effect is still largely debated in the literature. The influence of the used alkali metal cations on the CO_2_RR activity and selectivity is generally attributed to the relatively high concentration of alkali cations in the outer Helmholtz plane (OHP). Early work from Monteiro et al. proposed that large cations are specifically adsorbed more easily on the catalyst surface because of the fewer coordinated water molecules.^117^ Adsorbed cations can also elevate the potential at the OHP and decrease the local proton concentration, suppressing the HER.^118^ Alternatively, it was suggested that the cation size can significantly affect the rate of water hydrolysis by tuning the hydration energy.^119^ For instance, the p*K*_a_ value of Li^+^ was calculated to be three times higher than that of Cs^+^. The hydrated Cs^+^ acts as a buffer, maintaining a locally low pH near the electrode and increasing the local CO_2_ concentration compared to Li^+^ by 28 times (Figure 9a). To gain more insight into the role of cations in electrocatalysis, Ringe et al. developed a combined ab initio/continuum model of cation and electric double layer field effects based on a continuum-modified Poisson–Boltzmann approach (Figure 9b).^120^ By applying a single set of cation sizes derived from experimental data, the model showed quantitative agreement with the experiments for the catalyst system on both Ag and Cu. Their theoretical model and experimental results indicate that the repulsive interactions derived from the hydrated cations in the Helmholtz layer should be responsible for the change of the surface charge and their electric field. The use of high-valent cations with a small hydration radius also increases the potential of zero charges or capacitance, which maximizes the surface charge density and the corresponding interfacial electric fields.^121^ Bell’s group provided insights regarding the beneficial effect of cations, particularly at relatively low overpotentials for which the reaction rate does not perturb the local pH.^122,123^ Notably, the hydrogen and CH_4_ partial currents remained steady, while formate, C_2_H_4_, and C_2_H_5_OH formation rates increased when using large alkali cations. The cation size-independent production of H_2_ and CH_4_ was attributed to the zero dipole moment of *H and *CHO, which are the corresponding reaction intermediates of the reactions (Figure 9c).

Alkali metal cations have also been used to promote the CO_2_RR in strongly acidic media. A key advantage to operating at a low pH is the improved carbon utilization efficiency, which is limited in neutral and alkaline media due to the formation of carbonate. Sargent and co-workers utilized a cation-augmenting layer to sustain a high K^+^ concentration at the copper catalyst surface.^107^ They achieved a Faradaic efficiency of 61% for CO_2_RR products and that of 40% for C_2+_ products at 1.2 A/cm^2^, and by lowering the CO_2_ flow they reached a single pass conversion efficiency of 77%. Gu et al. explored the effect of alkali cations on the CO_2_RR in acid with tin oxide, gold, and copper catalysts, achieving 90% Faradaic efficiencies for formic acid and CO.^124^ Using a simulation based on the Poisson–Nernst–Planck (PNP) model, they predicted that the origin of such striking effects was the modulation of electric fields, which inhibited the migration of hydrononium ions.

### 4.3 The Search for Novel Electrolytes: Ionic Liquids and Nonaqueous Electrolytes

Ionic liquids (ILs), which are defined as salts that remain liquid below 100 °C, have been proven to be a promising new class of environmentally benign solvents.^125^ By tuning the molecular structure and polarity of the IL, the CO_2_ and N_2_ absorption capacity and the ability to stabilize charged CO_2_ and N_2_ species can be tuned and optimized. ILs also possess several advantages, such as a wide electrochemical windows, thermal and chemical stability, negligible volatility, and electron transfer mediation for redox catalysis, which make them interesting alternatives for promoting the CO_2_RR and NRR.^126^ As they are nonaqueous by nature, ILs allow control of the aqueous content to an optimum level to provide protons for hydrocarbon formation while suppressing the HER.^127−131^

**Figure 10:**
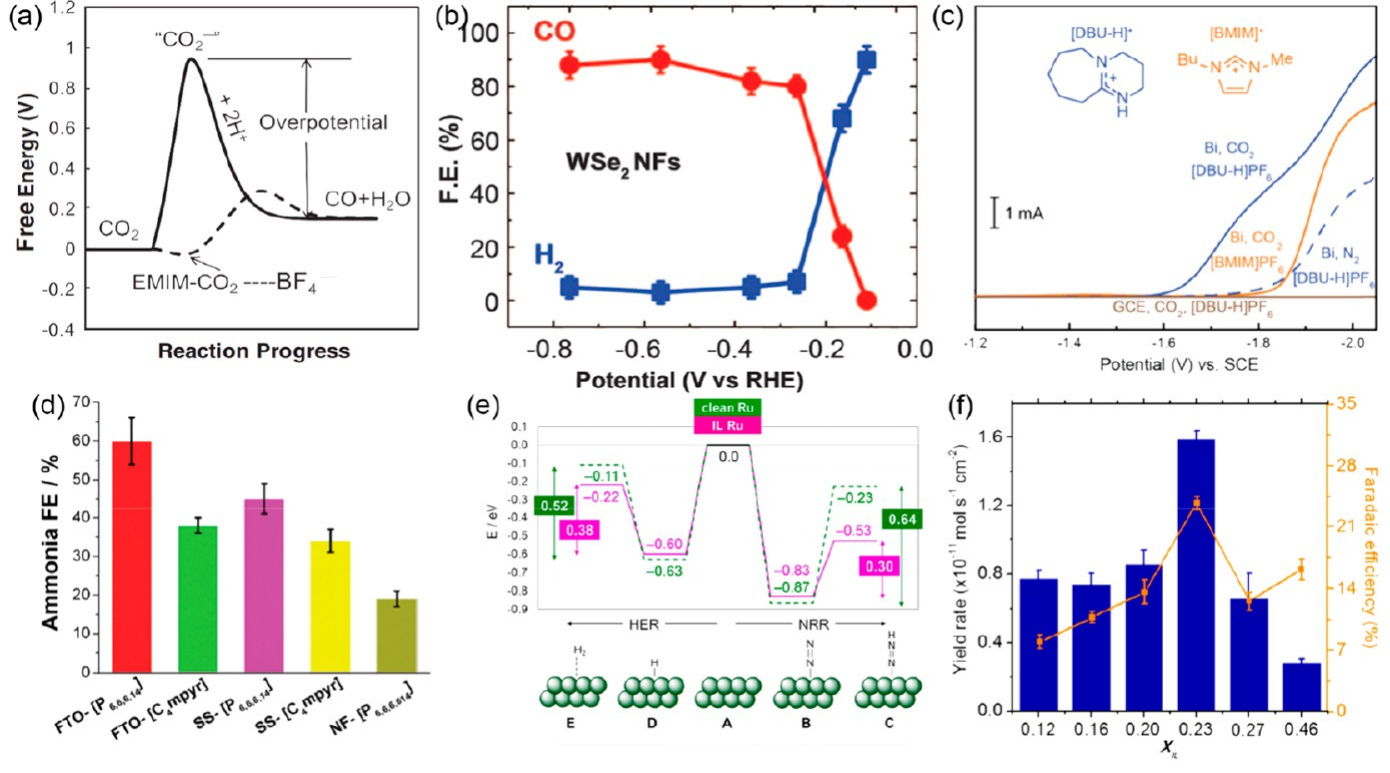
(a) Schematic of how the free energy of the system changes during the CO_2_ + 2H^+^ + 2e^–^ ⇌ CO + H_2_O reaction in water, acetonitrile (solid line), or EMIM-BF_4_ (dashed line). Reproduced with permission from ref (132). Copyright 2011 AAAS. (b) Overall FE_CO_ and FE_H2_ at different applied potentials for WSe_2_ NFs. The error bars represent the standard deviation of four measurements. Reproduced with permission from ref (134). Copyright 2016 AAAS. (c) Linear sweep voltammograms were recorded for Bibased and bare GCEs in MeCN containing 250 mM IL and 0.1 M TBAPF_6_ under the saturation of Ar, N_2_, or CO_2_. Reproduced with permission from ref (137). Copyright 2018 American Chemical Society. (d) Faradaic efficiency for the electroreduction of N_2_-saturated ILs on various electrodes at a constant potential of 0.8 V vs. NHE. Reproduced with permission from ref (139). Copyright 2017 Royal Society of Chemistry. (e) Corresponding reaction energy profiles of such intermediates during the NRR (right) and HER (left) for clean (dashed green line) and IL-decorated (solid purple line) Ru surfaces. Reproduced with permission from ref (140). Copyright 2019 American Chemical Society. (f) Solvent/IL ratio (*X*_IL_) dependence of the NH_3_ yield and FE at −0.65 V vs NHE. Reproduced with permission from ref (141). Copyright 2018 American Chemical Society.

ILs have been extensively investigated for the CO_2_RR because the cations of ILs can form a complex with CO_2_ and further activate it. Rosen et al. reported the use of 1-ethyl-3-methylimidazolium tetrafluoroborate (EMIM-BF_4_) as an IL electrolyte for the electrochemical conversion of CO_2_ to CO on silver (Figure 10a).^132^ The IL system lowers the energy of the *CO_2_ intermediate via the formation of a complex intermediate, which lowers the energy associated with the initial step of the reduction reaction.^133^ The formation of CO occurred at a very low onset overpotential, and the IL system demonstrated sustained production of CO for 7 h with a FE_CO_ of more than 96%. ILs have also been applied with transition metal dichalcogenides, which are known to be more prone to promote the HER over other reduction reactions. Remarkably, Asadi et al. exfoliated WSe_2_ nanoflakes to perform the electroreduction of CO_2_ to CO using a 50 vol % [Emim]BF_4_/H_2_O solution.^134^ The current density, FE, and TOF in the production of CO were all superior at lower overpotentials, suggesting a high selectivity for the CO_2_RR (Figure 10b). Copper selenide nanocatalysts have been identified to convert CO_2_ to CH_3_OH at low overpotentials in a [Bmim]PF_6_/acetonitrile/H_2_O mixed electrolyte.^135^ In addition, in a [Bmim]BF_4_/H_2_O electrolyte, MoTe_2_ could also be used as a catalyst for CO_2_ reduction to CH_4_ with a high FE of 83% at a relatively low overpotential.^136^ Atifi et al. demonstrated that protic ionic liquids (PILs) derived from 1,8-diazabicyclo[5.4.0]undec-7-ene (DBU) effectively promote the electrochemical reduction of CO_2_ to formate (HCOO^–^) with high selectivity (Figure 10c).^137^ The use of PILs composed of the conjugate acid of DBU, [DBU-H]^+^, efficiently catalyzed the reduction of CO_2_ to HCOO^–^ (FE_HCOOH_ ≈ 80%) with significant suppression of CO and H_2_ production (FE_CO_ + FE_H2_ ≈ 20%) in either acetonitrile or an acetonitrile/H_2_O mixed electrolyte.

Ionic liquids and nonaqueous electrolytes with high N_2_ solubility under ambient conditions can also increase the local concentration of N_2_ near the catalyst surface by as much as 20 times compared to water on a volumetric basis.^138^ MacFarlane and co-workers reported the use of ionic liquids with high N_2_ solubility for the electroreduction of N_2_ to ammonia at room temperature and atmospheric pressure.^139^ As presented in Figure 10d, a FE_NH3_ as high as 60% was achieved in [P6,6,6,14][eFAP]. Ortuño et al. used DFT calculations to explore the nature of N_2_ adsorption on different ions and found that a stronger interaction accompanied by charge delocalization will result in stronger adsorption of N_2_.^140^ As shown in Figure 10e, they found that on a Ru surface the presence of ILs reduces the relative electronic energy of the N_2_RR intermediate N_2_H* more significantly than that of the HER intermediate, H_2_*, lowering the energy by 0.34 and 0.11 eV, respectively. Suryanto et al. identified the impact of the IL molar fraction (*X*_IL_) on the physicochemical properties of the electrolyte mixture and the NRR performance.^141^ A FE as high as 23.8 ± 0.8% with an NH_3_ yield rate of 1.58 ± 0.05 × 10^–11^ mol/s·m^2^ was achieved for *X*_IL_ = 0.23 at an optimal potential of −0.65 V vs. NHE (Figure 10f). Note that in this study, which predates the publication of standard NRR protocols mentioned in the above text, no ^15^N labeling studies were provided, but an extensive purification of the N_2_ reactant was carried out. The significant drop in the NRR performance when further increasing *X*_IL_ highlights the role of 1*H*,1*H*,5*H*-octafluoropentyl 1,1,2,2-tetrafluoroethylene ether (FPEE) in facilitating the mass transport of N_2_ in the electrolyte. The authors suggested that other factors correlating FE and X_IL_ could play a role, such as the presence of complex molecular interactions and the different diffusion behaviors of neutral N_2_ molecules and polar H_2_O within the mixed electrolyte system, a known phenomenon with ionic liquids.^142^

### 4.4 Solid-State Electrolyte Designs

**Figure 11:**
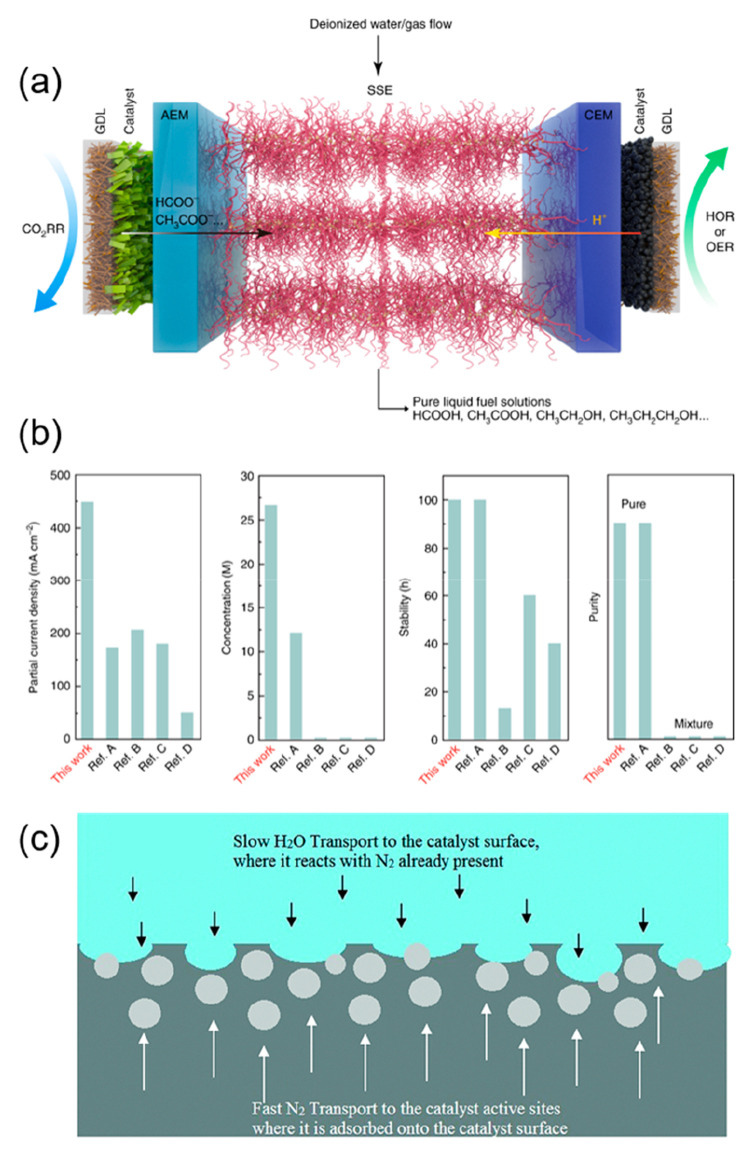
(a) Schematic illustration of the CO_2_ reduction cell with a solid electrolyte. Reproduced with permission from ref (147). Copyright 2019 Nature Publishing Group. (b) Electrochemical performance of an all-solid-state CO_2_RR reactor compared with previous literature. Reproduced with permission from ref (148). Copyright 2020 Springer Nature. (c) Cathode species transport diagram illustrating the advantage of the polymer gel electrolyte to limit water transport. Reproduced with permission from ref (149). Copyright 2018 Royal Society of Chemistry.

Conventional liquid electrolytes used in the CO_2_RR and NRR, such as KHCO_3_, Na_2_SO_4_, or KOH, mainly have three purposes: (i) to transport ions between the cathode and anode for efficient current flow, (ii) to provide protons for successive PCET, and (iii) to solvate liquid products. The mixture of liquid products and ion impurities requires energy- and cost-intensive downstream separation steps to obtain pure products, which complicate the infrastructure for delocalized production.^143^ To tackle this problem, the concept of solid-state electrolytes was proposed, inspired by the progress in solid-state electrolytes for batteries.^144^ A solid-state electrolyte is typically placed between ion-exchange membranes with close contact to efficiently transport the generated ions and minimize the ohmic loss of the device.^145^ Remarkably, solid-state electrolytes were found to be very effective in suppressing the HER by limiting the flow of protons to the catalyst active sites during the electrochemical CO_2_RR.^146^ The Wang group reported the continuous electrocatalyic conversion of CO_2_ to pure liquid fuels using two-electrode systems with solid electrolytes.^147,148^ They applied a porous solid electrolyte (PSE) layer composed of styrenedivinylbenzene copolymer microspheres with sulfonic acid functional groups for proton conduction. Using a formic acid-selective bismuth catalyst (FE_HCOOH_ ∼ 97%), the electrochemically generated protons and formate anions could combine at the PSL to produce formic acid (Figure 11a). By directly flowing a carrier gas instead of deionized water through the PSL, the authors were able to collect product vapors that could be condensed to form the pure product (almost 100 wt % formic acid), alongside an impressive current density and stability (Figure 11b).

Sheets et al. proposed a novel polymer gel approach to convert N_2_ to NH_3_ at mild temperatures (30–60 °C) and pressures (20 psig).^149^ As illustrated in Figure 11c, the polymer gel electrolyte helped to control the rate of the HER by limiting water transport and boosting N_2_ transport, thus improving the selectivity toward the NRR.

## 5 Three-Phase Interface Engineering

The abundance of protons near the catalyst active sites constitutes a significant challenge for the catalyst selectivity vs the competing HER in aqueous electrolytes, resulting in low selectivity and activity for the CO_2_RR and NRR. A mitigation strategy resides in facilitating the accessibility of the catalyst to high concentrations of CO_2_ or N_2_ molecules. While protons (H^+^) are readily available in aqueous solutions via water ionization, the supply of CO_2_ and N_2_ molecules to the catalyst surface is limited by their low concentration and slow diffusibility. In saturated aqueous electrolytes, the solubility of CO_2_ in H_2_O is 33 mmol/L at 298 K and 1 atm pressure, whereas the value for N_2_ in H_2_O remains as low as 0.7 mmol/L.^19^ By comparison, the concentration of protons in a neutral aqueous electrolyte is typically 2.7-fold and 132-fold higher than the concentrations of CO_2_ and N_2_, respectively.

In the context of CO_2_RR, Raciti et al. demonstrated that the local concentration of CO_2_ at the catalyst surface can reach zero under strong reaction driving force conditions, hence lowering the selectivity by limiting the supply of the reactant.^150^ Significant advances to minimize this reactant supply issue at the electrode have been made thanks to the implementation of efficient three-phase interfaces between gaseous CO_2_, the liquid electrolyte, and the solid catalyst. The most typical realization of such a three-phase interface involve porous gas diffusion layer (GDL) electrodes, which allow the delivery of gas-phase CO_2_ directly to the catalyst active sites. Such a strategy, resulting in higher CO_2_ and lower H^+^ surface concentrations, has the potential to improve CO_2_RR performances while significantly lowering competitive HER. The properties of the GDL can affect CO_2_ and water transport heavily, and main advances in this field have been recently reviewed^151,152^ and will not be extensively reviewed here in the context of CO_2_RR. Thinner GDL/catalyst layers shorten the CO_2_ diffusion distance, raising the relative CO_2_ concentration; however, excessively high concentrations can decrease multicarbon product formation by competing with intermediates such as CO for binding sites. Tan et al. found that by adjusting the catalyst layer structure and the CO_2_ feed concentration and flow rate they could establish a moderate local CO_2_ concentration that was optimal for multicarbon product selectivity.^153^

**Figure 12:**
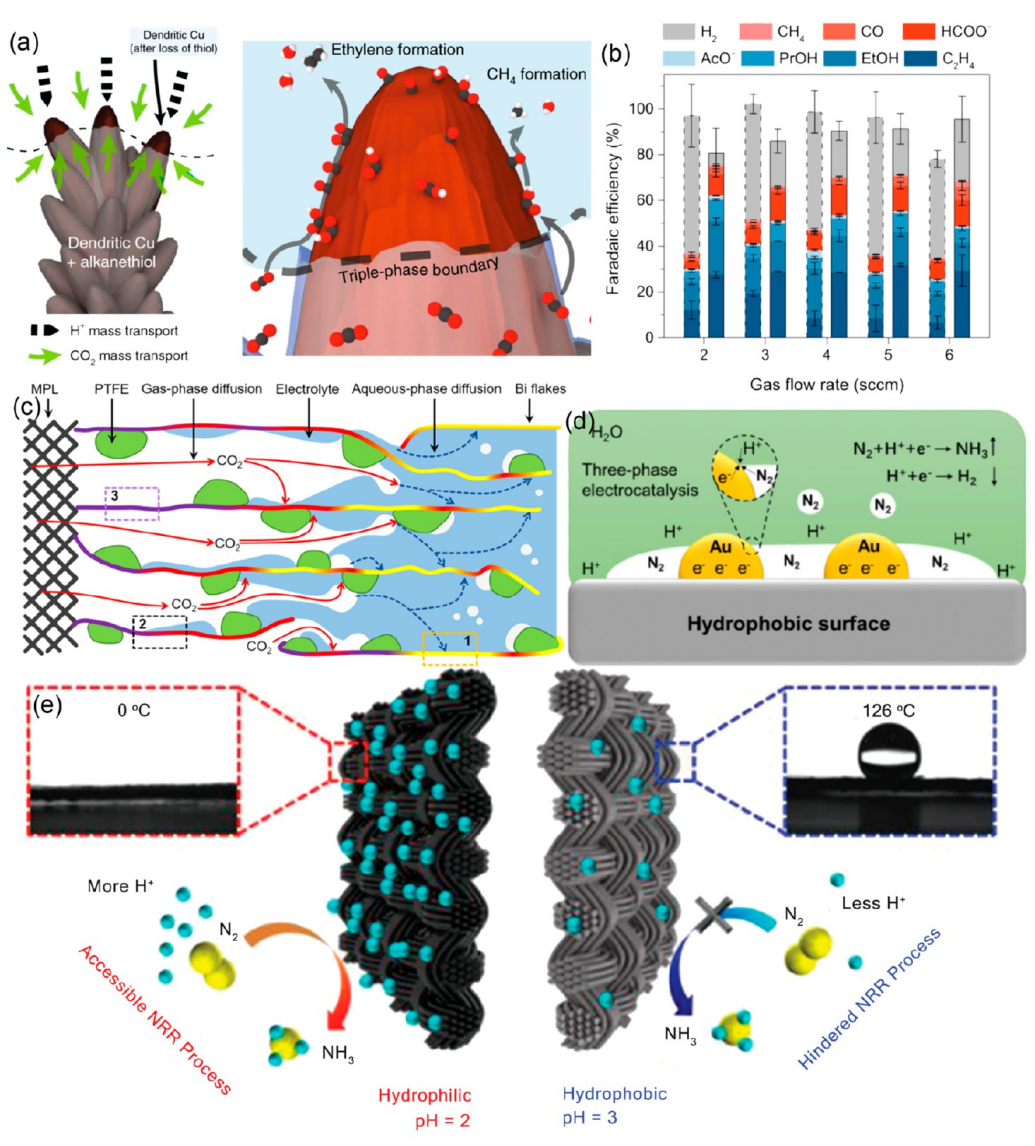
(a) Operation of the hydrophobic dendrite, illustrating the enhanced CO_2_ mass transport from the triple-phase boundary between the electrolyte, the electrode, and gaseous CO_2_ and the resultant formation of key products on the surface. Reproduced with permission from ref (22). Copyright 2019 Springer Nature. (b) Faradaic efficiencies for the CO_2_RR on the two electrodes (dashed, AvCarb MGL370 + Cu/C; solid, AvCarb GDS2230 + Cu/C) at −1.0 V vs. RHE with various CO_2_ flow rates. Reproduced with permission from ref (155). Copyright 2021 Springer Nature. (c) Schematic illustration of CO_2_ mass transport inside the catalyst layer with added PTFE, including gas-phase diffusion (solid red arrows) and aqueous-phase diffusion (dashed blue arrows). The dashed rectangles indicate catalyst areas that are only exposed to the electrolyte, thoseexposed to both electrolyte and gaseous CO_2_, and those only exposed to gaseous CO_2_. Reproduced with permission from ref (156). Copyright 2021 American Chemical Society. (d) Schematic illumination of the three-phase contact for N_2_ (gas), the electrolyte (liquid), and the catalyst (solid) at the hydrophobic interface. Reproduced with permission from ref (161). (e) NRR catalytic mechanism of Mo_2_C/C under proton-suppressed and proton-enriched conditions. Reproduced with permission from ref (162). Copyright 2018 Wiley.

Alternatively to requiring GDL-based electrodes, the catalyst support itself can be modulated to modulate the three-phase interface via a fine-tuning of the local microenvironment near the catalyst surface through nanostructuring and surface functionalization. Inspired by biological strategies to entrap a gas layer at the surface of a solid, and in particular by the plastron effect enabling the diving bell spider to breathe underwater, Wakerley et al. functionalized porous dendritic Cu electrodes generated via the DHBT strategy mentioned above in section 3 with long-chain alkanethiols. The resulting superhydrophobic Cu electrodes demonstrated a sixfold decrease of HER upon treatment with the alkanethiol and a subsequent drastic increase in CO_2_ reduction selectivity.^22^ They proposed that the hydrophobicity establishes triple-phase interfaces at the electrode where CO_2_ mass transport is omnidirectional and H^+^ mass transport is unilateral (Figure 12a). This increases the local CO_2_ concentration and thereby the surface concentration of Cu-COOH* and Cu-CO*, enhancing C–C coupling. This study led to the identification of the role of hydrophobicity and the formation of gaseous voids as effective levers to orient the reaction pathway toward the formation of multicarbon products. Khan and co-workers explored the idea of gas-trapping further using a gasphilic silicon substrate in proximity to the catalyst layer. Creating a CO_2_ plastron adjacent to the catalyst improved mass transfer, enriching and maintaining the local CO_2_ concentration. Using a smooth copper catalyst, they recorded improved activity and a decrease in FE_H2_ (13% compared to 29% with bulk CO_2_ bubbling). These trends were replicable using nanostructured copper, demonstrating the transferability of such an approach to different catalysts.^154^ Moreover, Xing et al. showed that a hydrophobic microenvironment can significantly enhance CO_2_ electrolysis by facilitating reactant diffusion (Figure 12b).^155^ Using commercial copper nanoparticles dispersed with hydrophobic polytetrafluoroethylene (PTFE) nanoparticles, they reported improved activity and Faradaic efficiency for CO_2_ reduction with a partial current density of >250 mA/cm^2^ and a single-pass conversion of 14% at moderate potentials. Importantly, this performance was approximately twice as large as that of regular electrodes without added PTFE. Similar findings were also observed from a Bibased catalyst modified with PTFE nanoparticles in the catalyst layer to demonstrate a partial current density of 677 mA/cm^2^ for formate and 35% single-pass CO_2_ conversion at −0.7 V vs. RHE (Figure 12c).^156^ Pham et al. compared various ionomeric binders on a Cu catalyst and achieved a 77% Faradaic efficiency and 600 mA/cm^2^ partial current density for C_2+_ products at −0.76 V vs RHE using a fluorinated ethylene propylene (FEP) binder.^157^ They attributed these results to the hydrophobic properties of FEP. The Sinton and Sargent groups have also done notable work on modulating the three-phase interface in continuous flow and membrane electrode assembly (MEA) electrolyzers, enabling high current densities (e.g., > 1 A/cm^2^) to be achieved.^103,146^ For example, they presented a catalyst:ionomer bulk heterojunction (CIBH) architecture that had both hydrophilic and hydrophobic functionalities. By having different domains that favored gas and ion transport routes, they were able to decouple gas, ion, and electron transport, extending the reaction interface from the submicrometer range to the several micrometer range.^103^ These examples illustrate that the moderate hydrophobicity of the catalyst layer can establish a microenvironment with a balance between gaseous CO_2_ and liquid electrolytes inside the catalyst layer. Such microenvironments—equivalent to microreactors—reduce the thickness of the diffusion layer, accelerate CO_2_ mass transport, and link highly active reaction zones at the interfaces between the three phases involved in the reaction.^158^ The triple-phase interface can also be further tuned by applying ionomers to control pH and CO_2_/H_2_O concentrations. Bell and co-workers postulated that anion-exchange ionomers (e.g., sustainion) increase CO_2_ solubility, cation-exchange ionomers (e.g., nafion) increase local pH by trapping OH^–^ ions, and both types increase water concentration.^159^ By optimizing a bilayer ionomer coating and coupling to pulsed electrolysis, they achieved a Faradaic efficiency of 90% for C_2+_ products and that of just 4% for H_2_.

In the case of NRR, when applying large potentials at the electrodes, the kinetically facile HER becomes preferable to the reduction of N_2_ due to the relatively low energy barrier associated with the reaction. It was suggested that the HER should always dominate at normal proton concentrations near the metal electrode surface. However, when few protons or electrons are provided, the NRR may preferentially occur, as recently observed experimentally. Designing a triple-phase interface for NRR can increase the local N_2_ concentration and improve *N_2_ adsorption while limiting the availability of protons by reducing contact with the electrolyte.^160^ Using this strategy, Zhang et al. realized triple-phase electrolysis via in situ fabrication of Au nanoparticles located on hydrophobic carbon fiber paper (Au/CFP) (Figure 12d).^161^ The hydrophobic carbon fibers facilitated the formation of three-phase contact points (TPCPs) for N_2_, the liquid electrolyte, and the Au NPs. Xiao et al. successfully modified the d-band structure of a self-supporting nanoporous Mo_4_P_3_ catalyst by capping with a fluorosilane hydrophobic layer.^73^ This approach aims at weakening the ability of the material surface to adsorb protons while simultaneously preventing the decrease of the amount of water available at the active sites, thus further lowering the competitive HER. This hydrophobic Mo_4_P_3_ material exhibits decent NRR performances, with a FE of 10.1% and an NH_3_ yield rate of 17.3 μg/h·cm^2^. According to Wang and co-workers, excessive suppression of the HER is not, however, beneficial to NRR activity, although it can lead to higher Faradaic efficiency (Figure 12e). A sharp decrease in the local concentration of protons does not benefit the NRR process, as protons are necessary for the successive PCET steps associated with the formation of ammonia. These investigations point out that although the release of hydrogen is a competitive reaction, protons are paradoxically essential to increase the ammonia yield rates.^162^

## 6 Conclusions and Perspectives

The industrial development of the CO_2_RR and NRR is currently plagued by low Faradaic and energy efficiencies. The successive PCET steps associated with the corresponding reaction intermediates increase the complexity and complicate the search for an ideal catalyst. In contrast, the simplicity of the HER mechanism and the abundant presence of protons in traditional electrolytes make the production of hydrogen a competitive and parasitic reaction that consumes a significant amount of electrons to the detriment of the fixation of CO_2_ and N_2_. Additionally, the selectivity toward a single product, particularly important in the context of CO_2_RR is a central point to be considered. Multiple strategies have shown promise but still require the elaboration of a robust and rational framework; they have demonstrated that optimal activity and selectivity can be obtained upon modulating thermodynamics and kinetics of the reaction. By engineering the catalyst, electrolyte, and reaction interface, three main strategies have been applied toward that goal (Table 2): (i) targeting a narrow distribution of molecularly defined active sites, (ii) increasing the reactant/proton ratio at the three-phase interface where the reaction takes place to lower the undesired formation of H_2_, and (iii) the stabilization and confinement of reaction intermediates in the electrode vicinity to favor the formation of multielectron reduction products.

**Table 2:** Reference Numbers of Key Examples of the Three Strategies for Enhanced Product Selectivity in Carbon Dioxide and Nitrogen Reduction Reactions and How They Are Implemented^a^

	strategy	(i) molecularly defined active sites	(ii) high local reactant concentration	(iii) stabilizing and confining intermediates
catalyst design	porous networks		31	33, 41, 46
	nanostructures (e.g., wires, films, needles etc.)		51, 52, 55	53
	surface functionalization	62, 63, 67		58, 59, 62, 67, 71
	control of crystal size, facet and spacing	74–76, 79		
	single-site engineering	83, 88–93		83
electrolyte engineering	adjusting local pH		103, 105–107, 109	108, 110
	alkali metal cation effects		107, 124	122
	ionic liquids		139, 141	132, 133, 140
	solid-state electrolyte		145–147, 149	
three-phase interface engineering	gas diffusion layer electrodes		152–154	
	gas trapping		22, 154–156, 73,161	
	utilizing ionomers		103, 157, 159	

a References related to CO_2_RR are in normal text, while references related to NRR are underlined.

The complexity of the parameters involved to address these challenges simultaneously further highlights the interest in combining experimental and theoretical approaches to guide the design of both catalysts and electrolyzers for the CO_2_RR and NRR. From this perspective, machine learning will help rapid screening of catalysts with high selectivity based on massive data in the silico database by focusing on near-optimal bond energy with adsorbates, such as *CO and *N_2_H. In addition, enabling a better understanding and control of the PCET steps, notably via the elaboration of a robust framework to link the relative contributions of charge transfer and protonation steps to overpotential and the distribution of surface species, will be key to further rationally improve electrocatalysts.

This Review illustrated several examples displaying industry-relevant performances in terms of selectivity and current densities, highlighting the potential of electrochemical approaches for the preparation of carbon- and nitrogen-containing molecules. However, for the CO_2_RR, many studies have been performed in alkaline or neutral media, resulting in carbonate formation in the electrolyte. This is detrimental to carbon utilization and energy efficiency, especially considering the energy that would be required to regenerate spent electrolyte. This problem has been considerably underestimated and overlooked for some time; however, an increasing number of studies over recent years have attempted to tackle this issue. Using acidic electrolyte prevents carbonate crossover to the anode and regenerates CO_2_ close to the cathode surface, improving carbon utilization efficiency. Naturally, this media poses challenges regarding hydrogen evolution, and the application of strategies covered in this review will be pivotal in overcoming this.

Moreover, most of the presented strategies introduced in the present Review enable the improvement of catalyst selectivity for a relatively short period of time but have not been investigated over industrially relevant time scales. Maintaining high selectivity for the CO_2_RR and NRR over long operation times remains the largest challenge to date, as rapid loss in activity and selectivity is observed for most of the systems reported. This notably results from the fact that in operation undesirable intermediates or poisonous byproducts preferably deposit on the catalyst surface and affect the catalysis process. This phenomenon may decrease the effective area of the electrocatalyst, accelerate cathodic degradation, and increase selectivity toward competitive HER. The demonstration of catalysts with ultralong stabilities of >5000 h constitutes in our view the last milestone to be reached in order to validate the industrial potential of the CO_2_RR and NRR.
